# 5-year minimum follow-up study of Surgical & Radiological outcomes of Thoracoscopic assisted Anterior Instrumented fusion compared to posterior hook and pedicle screw instrumentation in the treatment of Adolescent Idiopathic Scoliosis

**DOI:** 10.1186/1748-7161-10-S1-O66

**Published:** 2015-01-19

**Authors:** Pankaj Kandwal, Hee-Kit Wong, Gabriel Liu, Zhen-Chang Liang

**Affiliations:** 1 All India Institute of Medical Sciences (AIIMS), Rishikesh, Uttarakhand, India; 2 University Spine Centre, National University Health System (NUHS), Singapore

## Objective

To compare surgical and radiological outcomes between Video assisted Thoracic Anterior Instrumentation (VATS), all Hooks/Hybrid, and all Pedicle Screw system with a minimum 5 yr follow-up.

## Material & methods

Patients with AIS Lenke 1, 2 curves undergoing selective thoracic fusion with minimum five year follow-up were included. Patients were divided into the following groups:

### Group 1

Thoracoscopic anterior instrumentation (VATS/VATI group) n=98; average age 14.4yrs.

### Group 2 &3

Posterior Instrumentation (PI): 91 patients, average age 14.2 yrs. The posterior group was divided into all Hook/hybrid (Group 2, n=45) and all Pedicle screw construct (Group 3, n=46), for evaluation of radiological outcomes. The groups were compared for various surgical and radiological outcomes at immediate post-op, 2 and 5 year intervals. Average follow-up 6.6 years (5-12 yrs).

## Results

The groups were similar with regards to age, Risser grade, and Cobb angle of Main Thoracic curve.

### Surgical outcomes

Mean blood loss was 382±348 ml in VATS/VATI group (Group1), which was significantly less when compared with Posterior Groups 2 and 3 with 780±386 ml (p=0.000). duration of surgery for Group 1 was 328±74 min, compared to the posterior groups at 236±53 min (p=0.000). Hospital stay in the VATS group was 7.45±4.9 days, which was significantly longer than the posterior groups (6.8±3.2; p value=0.000). Levels of instrumentation and number of screws per patient in Group 1 were 7.7±0.6 and 7.7±0.6 respectively; which were significantly less (p=0.000) compared to the posterior groups (10±1.8 and 12.21±2.37 respectively).

### Radiological outcomes

Immediate correction of main thoracic (MT) curve in Group 1 was 78.8% which was similar to Group 3 at 79.6%; & was significantly better than Group 2 at 56.7% (p< 0.05). The percentage correction of MT curve at the end of 2 and 5 year follow-up in Group 1 (77.4 &76.5%) was similar to Group 3 (76.3% and 73.7%); but was significantly better (p<0.05) than Group 2 (53.1% and 51.6%) respectively. There were 18 complications in Group 1 & 27 complications in Group 2 & 3. No deep wound infection was observed in Group 1.

## Conclusion

VATS and posterior instrumentation both achieved excellent correction of the main thoracic curve. The correction achieved by thoracoscopic and posterior pedicle screw system was well maintained at 5 years after surgery, while loss of correction occurred with posterior hook/hybrid constructs. VATS had advantages of fusing significantly less segments, less blood loss, and no deep wound infection. VATS was associated with longer operative time and longer hospital stay compared to posterior instrumentation.

